# Does Blue Uniform Color Enhance Winning Probability in Judo Contests?

**DOI:** 10.3389/fpsyg.2018.00045

**Published:** 2018-01-30

**Authors:** Peter D. Dijkstra, Paul T. Y. Preenen, Hans van Essen

**Affiliations:** ^1^Department of Biology, Central Michigan University, Mount Pleasant, MI, United States; ^2^Department of Sustainable Productivity and Employability, TNO, The Netherlands Organisation for Applied Scientific Research, Leiden, Netherlands; ^3^Judoinside.com, Veenendaal, Netherlands

**Keywords:** competition outcome, color, psychological functioning, human performance, contests, judo

## Abstract

The color of an athlete's uniform may have an effect on psychological functioning and consequently bias the chances of winning contests in sport competition. Several studies reported a winning bias for judo athletes wearing a blue outfit relative to those wearing a white outfit. However, we argue there is no winning bias and that previous studies were confounded and based on small and specific data sets. We tested whether blue biases winning in judo using a very extensive judo data set (45,874 contests from all international judo tournaments between 2008 and 2014). In judo, the first called athlete for the fight used to wear the blue *judogi* but this was changed to the white *judogi* in 2011. This switch enabled us to compare the win bias before and after this change to isolate the effect of the color of the *judogi*. We found a significant win bias for the first called athlete, but this effect was not significantly related to the color of the *judogi*. The lack of a significant win effect of *judogi* color suggests that blue does not bias winning in judo, and that the blue-white pairing ensures an equal level of play. Our study shows the importance of thoroughly considering alternative explanations and using extensive datasets in color research in sports and psychology.

## Introduction

The color of an athlete's uniform may bias the chances of winning contests in sport competition (Elliot and Maier, 2014). This is relevant to evolutionary psychologists interested in understanding how color influences performance in competitive contexts as well as sport policy makers who want to ensure an equal level of play. Specifically there is evidence for a win effect for athletes wearing red in combat (Hill and Barton, 2005) and team sports (Attrill et al., 2008; Piatti et al., 2012; but see Allen and Jones, 2014). These “red” effects seem both empirically and theoretically grounded. They can be attributed to the inherently intimidating effects of redness on opponents (Ten Velden et al., 2012; Elliot and Maier, 2014) and the link between wearing red and increased heart rate, physical strength and higher testosterone levels. There is also experimental evidence that red coloration itself enhances winning in many vertebrate species, including nonhuman primates (Baube, 1997; Pryke et al., 2001; Dijkstra et al., 2005; Khan et al., 2011) but see (Pollet and Peperkoorn, 2013).

Several studies suggested the color effects on winning are not unique to red (e.g., Rowe et al., 2005). Rowe et al. (2005) found in a study that judo players in blue won more often than those in white during the 2004 Olympics. They suggested that the effect may be a consequence of perceptual differences between blue and white color. The white *judogi* was suggested to be more visible than the blue *judogi*, allowing the athlete in blue to better evaluate and anticipate the movement of his (white) opponent. While perceptual differences in moving objects of a certain color may indeed influence localizing team players and potentially the likelihood of winning in for example football matches (Olde Rikkert et al., 2015), differential perception effects are unlikely to occur in situations where athletes are directly fighting an opponent in close quarters. A second explanation was given by Barton and Hill (2005). They attributed the winning effect of blue to the fact that blue has an intimidating effect on opponent. However, this is also very unlikely because while this psychological effect has been suggested for red coloration, blue coloration is not known to be an inherently intimidating color.

Interestingly, Dijkstra and Preenen (2008) showed that the Rowe et al. (2005) study in *Nature* was confounded by several alternative factors, namely allocation biases due to the seeding and ranking system, asymmetries in prior experience in the repechage, and differences in recovery time between contests. After excluding the repechage matches and matches involving a seeded athlete no bias was found in the 2004 Olympics. Additionally, analysis of 501 finals of 71 major judo tournaments (including Olympics, World, and European Championships) revealed no winning bias of players in blue after controlling for the confounding factors.

However, several researchers still believe in a blue winning bias in judo. Reports have either shed doubt on Dijkstra and Preenen's study (Sorokowski and Szmajke, 2011) or found that blue won more often than those dressed in white without adequately controlling for potentially confounding variables (Matsumoto et al., 2007; Julio et al., 2015). Matsumoto et al. (2007) found a strong blue bias in winning in four major international tournaments during the early 2000s but ignored five major tournaments (including Olympic and World Championships) in almost the same time frame with no blue bias. Clearly, it appears that most studies claiming that blue enhances winning are not only confounded, but also use very small and specific data sets. More studies are needed, preferably using extensive data sets and an experimental approach, to test whether blue biases winning in combat sports such as judo.

**Figure 1:**
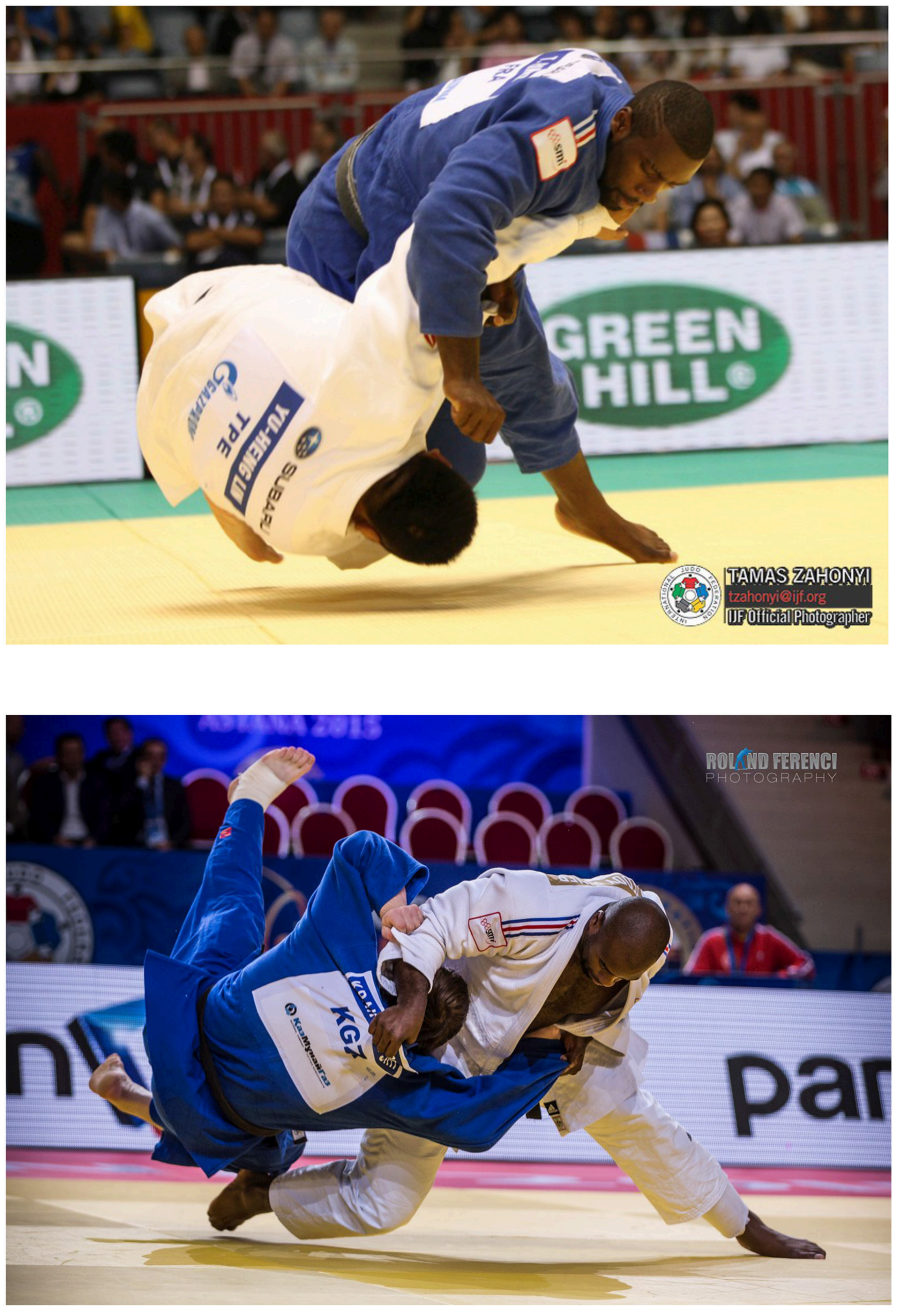
Shown here is heavyweight champion Teddy Riner in blue at the top and in white at the bottom at the World Championships in Tokyo (2010) and at the World Championships in Astana (2015), respectively. The first called athlete for the fight used to wear the blue *judogi* and the second called athlete the white *judogi*. However, starting at the World Championships in 2011 (WC 2011), the first called athlete wears the white *judogi*. Everything else remained the same. As a consequence, before the switch was introduced seeded athletes (such as Teddy Riner) mostly fought in blue, but are now mostly fighting in white. In addition, before the switch the athlete in blue had on average a longer recovery time between contests and was always to the right relative to the scoring board. After the switch, all this was reversed with now white having on average a longer recovery time and being placed to the right relative to the scoring board. Photo credit: IJF.org (top photo), and Judoinside.com (bottom photo). Reproduced with permission.

In judo, the first called athlete for the fight used to wear the blue *judogi* and the second called athlete the white *judogi* (Figure 1). However, starting at the World Championships in 2011 (WC 2011), the first called athlete wears the white *judogi*. This switch was introduced by the International Judo Federation (IJF) and the European Judo Union (EJU) to reflect the importance of the traditional white *judogi* (see “EJU takes over “white *judogi* first” change”: http://www.eju.net/news?mode=showNewsItem&id=1159&portalId=21). Everything else remained mostly the same. As a consequence, before the switch was introduced seeded athletes fought in blue, but are now fighting in white. In addition, before the switch the athlete in blue had on average a longer recovery time between contests but after the switch the athlete in white had on average a longer recovery time between contests. Finally, before the switch the athlete in blue was to the right relative to the scoring board and the referee but after the switch the athlete in white took over that position.

This switch enables us to compare the win bias before and after this change to isolate the effect of the color of the *judogi*. We analyzed contest outcomes in over 45,000 contests in major international judo tournaments between 2008 and 2014 to test whether blue increases the likelihood of winning. This large dataset overcomes some of the statistical limitations of other studies where only a single tournament or a small subset of tournaments or rounds were analyzed. We hypothesize that there is a significant win bias for the *first called athlete* (blue before WC 2011, white after WC 2011), but no significant effect of *judogi* color.

## Materials and methods

We developed an extensive database using information from the IJF and the EJU published on Judoinside.com. Our database included Olympic Championships, World Championships, World Cups, Grand Prix, Grand Slams, and European Championships. Our database comprised 162 tournaments, 45,874 contests in 14 weight categories (seven for both women's and men's division). In some tournaments, there is an open weight category; in this non-Olympic category, athletes from all weight categories can compete. We excluded this category in our analysis, because we expect substantial ability asymmetries between athletes due to large weight differences, obscuring any potential color effect on winning. We also excluded the repechage due to asymmetries in prior experience in relation to the first and second called athlete.

Statistical analyses were implemented in R version R3.2.1. We analyzed the women's and men's divisions separately. We used χ^2^-tests to compare the number of contests won and lost by the first called athlete. We tested whether the probability of the first called athlete winning the fight was dependent on the *judogi* color of the first called athlete. We refer to tournaments where the first called athlete for the fight wore a blue *judogi* (until WC 2011) “first called athlete blue”; tournaments where the first called athlete for the fight wore a white *judogi* (after WC 2011) are referred to as “first called athlete white.” We ran a mixed effects logistic regression model with first athlete color (referred to as “color switch”) and “round” (1/32, 1/16, 1/8, ¼, ½, final) as fixed variables, and tournament (referred to as “event”) and identity of first athlete (referred to as “judoka”) as random effects. The integer scalar (nAGQ)—the number of points per axis for evaluating the adaptive Gauss Hermite approximation to the log-likelihood was set to 1, corresponding to the Laplace approximation (Bates et al., 2015). Models were run using the *lme4* package (Bates et al., 2015). We report fixed effect estimates with 95% confidence intervals. Model summaries were generated using the *sjPlot* package (Ludecke, 2016). Tests of significance were two-tailed.

## Ethics statement

The current study has been reviewed by the director of the Research Compliance Office, Central Michigan University. Following the definition of the U.S. Department of Health & Human Services, the current study does not involve human subjects and our study was therefore not subject to review by the Institutional Review Board of Central Michigan University.

## Results

**Figure 2:**
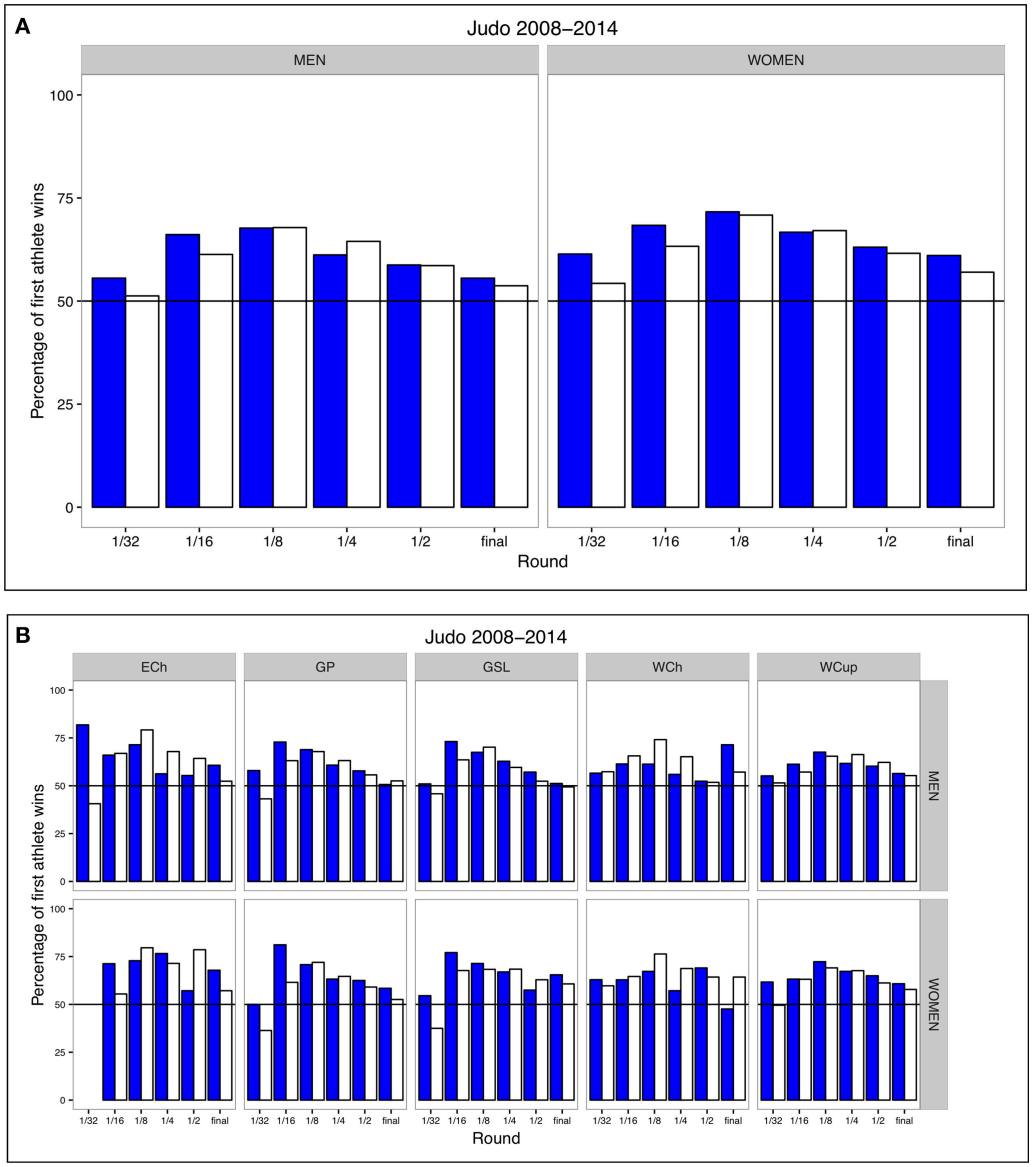
**(A)** Influence of *judogi* color on the outcome of judo contests. Shown in blue is the percentage of contests won by the first called athlete wearing blue (all tournaments until the World Championships of 2011); shown in white is the percentage of contests won by the first called athlete wearing white (all tournaments after the World Championships of 2011). Data of all contests (*n* = 45,874) were combined to calculate the percentage of wins of the first called athlete for each weight category. **(B)** Influence of *judogi* color on the outcome of judo contests for each tournament type separately. Shown in blue is the percentage of contests won by the first called athlete wearing blue (all tournaments until the World Championships of 2011); shown in white is the percentage of contests won by the first called athlete wearing white (all tournaments after the World Championships of 2011). Data of all contests (*n* = 45,874) were combined to calculate the percentage of wins of the first called athlete for each weight category.

Across all rounds there was an expected win bias for the first called athlete both before (first called athlete wearing blue) and after (first called athlete wearing white) the World Championships of 2011 when the *judogi* color of the first called athlete was changed (Figure 2).

If the blue *judogi* caused a win bias, the winning probability of the first called athlete would be predicted to be higher before the color switch compared to after the switch. However, in the men's division, the winning probability of the first called athlete was not statistically different between contests that took place before and those that took place after the color switch of the first called athlete (effect of color switch using mixed effects logistic regression, see Table 1A). In other words, the switch from a blue to a white *judogi* for first called athletes was not related to chances of winning. We repeated the same analysis for each tournament type separately and found no significant effects of the color switch except for the Grand Prix tournaments (Table 1B). A closer examination of the data suggests that this is mostly driven by a stronger probability of the first called athlete winning prior to the color change in round 1/32 and 1/16 compared to after the color change, possibly due to an increased effect of seeds resulting from a lower level of competition before the color change relative to after the color change. Excluding round 1/32 and 1/16 resulted in no significant effect of the color switch (mixed effects logistic regression, effect of color switch on the probability of first athlete winning. Odds ratio: 1.01, 95% [0.82–1. 24], *p* = 0.942, *N* = 3,515).

**Table 1A:** Effect of the color switch on the probability that the first athlete wins.

	**Men's division**			**Women's division**		
	***OR***	***CI***	***p***	***OR***	***CI***	***p***
**FIXED PARTS**						
(Intercept)	0.42	0.35–0.50	<**0.001**	0.45	0.36–0.57	<**0.001**
Color switch	1.04	0.90–1.20	0.606	1.19	0.96–1.47	0.114
Round (1/32)	3.00	2.52–3.58	<**0.001**	3.85	2.90–5.11	<**0.001**
Round (1/16)	3.28	2.83–3.81	<**0.001**	4.06	3.40–4.85	<**0.001**
Round (1/8)	2.99	2.59–3.44	<**0.001**	3.66	3.13–4.28	<**0.001**
Round (1/4)	1.96	1.70–2.26	<**0.001**	2.24	1.93–2.61	<**0.001**
Round (1/2)	1.36	1.17–1.59	<**0.001**	1.42	1.21–1.67	<**0.001**
**RANDOM PARTS**						
^τ^00, Judoka	0.876			1.245		
^τ^00, Event	0.161			0.380		
N_Judoka_	3,055			1,787		
N_Event_	162			162		
ICC_Judoka_	0.203			0.253		
ICC_Event_	0.037			0.077		
Observations	27,384			18,490		
AIC	34,597.43			22,400.26		
Deviance	30,842.23			19,535.52		

*Effect of the color switch on the probability that the first athlete wins (first called athlete wears blue is used as reference category) for the men's (left) and the women's (right) division using mixed effects logistic regression. Also shown are the effects of round (compared to the final) and the random effects of judoka and tournament (event). For fixed effects, we report the odds ratio [with its 95% confidence interval and significance level (p < 0.05) shown in bold]. Shown are the within-group variance (σ^2^), the between-group variance (τ_00_), the sample size of the random effects (event and judo athlete), and the intraclass coefficient correlation (ICC). The sample size (observations) as well as the Akaike Information Criterion and the deviance of the model are also indicated*.

**Table 1B:** The Effect of the color switch on the probability that the first athlete wins or each tournament type separately.

**Men's division**	**European championships**			**Grand prix**			**Grand slams**			**World championships and olympic games**			**World cups**		
	***OR***	***CI***	***p***	***OR***	***CI***	***p***	***OR***	***CI***	***p***	***OR***	***CI***	***p***	***OR***	***CI***	***p***
**FIXED PARTS**															
(Intercept)	0.75	0.38–1.47	0.401	0.71	0.52–0.98	**0.036**	0.51	0.34–0.77	**0.001**	0.53	0.26–1.07	0.075	0.75	0.61–0.92	**0.005**
Color switch	1.11	0.86–1.43	0.442	0.83	0.71–0.97	**0.019**	0.86	0.68–1.09	0.211	1.12	0.92–1.38	0.264	1.08	0.96–1.22	0.196
Round (1/32)	2.01	0.85–4.75	0.112	1.56	1.07–2.27	**0.022**	2.20	1.41–3.44	**0.001**	2.18	1.08–4.40	**0.030**	1.65	1.30–2.08	<**0.001**
Round (1/16)	2.51	1.27–4.99	**0.008**	3.10	2.27–4.22	<**0.001**	4.71	3.19–6.94	<**0.001**	2.43	1.22–4.85	**0.012**	1.85	1.51–2.26	<**0.001**
Round (1/8)	3.39	1.72–6.70	<**0.001**	2.99	2.22–4.04	<**0.001**	3.66	2.53–5.30	<**0.001**	2.18	1.09–4.38	**0.028**	2.23	1.84–2.70	<**0.001**
Round (1/4)	1.57	0.79–3.13	0.200	1.97	1.46–2.67	<**0.001**	2.17	1.49–3.16	<**0.001**	1.25	0.61–2.57	0.539	1.76	1.45–2.15	<**0.001**
Round (1/2)	1.23	0.59–2.59	0.582	1.34	0.97–1.85	0.075	1.40	0.94–2.10	0.098	0.64	0.29–1.39	0.258	1.38	1.12–1.70	**0.003**
**RANDOM PARTS**															
^τ^00, Judoka	0.479			0.492			0.657			0.992			0.488		
^τ^00, Event	0.000			0.012			0.048			0.000			0.035		
N_Judoka_	430			1,203			981			845			2,545		
N_Event_	7			36			24			7			88		
ICC_Judoka_	0.13			0.13			0.16			0.23			0.13		
ICC_Event_	0.00			0.003			0.01			0.00			0.01		
Observations	1,369			5,736			4,576			2,368			13,335		
AIC	1,714.13			7,341.37			5,781.96			3,054.97			1,7396.24		
Deviance	1,500.45			6,562.05			5,022.87			2,436.00			1,5612.28		

*For fixed effects, we report the odds ratio [with its 95% confidence interval and significance level (p < 0.05) shown in bold]. Shown are the results for the men's division*.

Our dataset included all major international tournaments between the year 2008 and 2012. Since color of the first called athlete was not randomly assigned across tournaments and years there is a possibility that the effect of the color switch was masked by potential changes in tournament format over the extended time frame of this study. To reduce this potential bias, we reanalyzed the dataset by focusing on tournaments between 2010 and 2012 only. Again, the effect of the color switch on the probability of first athlete winning was not significant (Odds ratio: 1.09, 95% [0.88–1.34], *p* = 0.439, *N* = 13, 834).

Similar to the findings in the men's division, in the women's division the *judogi* color switch did not significantly change the winning probability of the first called athlete (effect of the color switch, see Table 1A). Similar results were found when the data was analyzed for each tournament type separately (Table 1C) or when focusing on tournaments that took place between 2010 and 2012 only (Odds ratio: 1.12, 95% [0.87–1.44], *p* = 0.384, *N* = 9,459). In all analyses, the final model included the identity of the first called athlete and tournament as random effects (Table 1).

**Table 1C:** The effect of the color switch on the probability that the first athlete wins for each tournament type separately.

**Women's division**	**European championships**			**Grand prix**			**Grand slams**			**World championships and olympic games**			**World cups**		
	***OR***	***CI***	***p***	***OR***	***CI***	***p***	***OR***	***CI***	***p***	***OR***	***CI***	***p***	***OR***	***CI***	***p***
**FIXED PARTS**															
(Intercept)	1.12	0.56–2.25	0.746	0.97	0.68–1.37	0.862	0.65	0.39–1.09	0.103	0.45	0.21–0.95	**0.036**	0.78	0.61–0.99	**0.044**
Color switch	0.94	0.67–1.32	0.731	0.88	0.69–1.13	0.315	1.13	0.73–1.74	0.581	1.19	0.81–1.75	0.374	1.14	0.93–1.40	0.196
Round (1/32)				0.78	0.24–2.51	0.676	1.20	0.47–3.10	0.704	2.77	1.26–6.10	**0.011**	2.16	1.46–3.20	**<0.001**
Round (1/16)	1.63	0.75–3.51	0.215	2.49	1.76–3.53	**<0.001**	5.08	3.20–8.05	**<0.001**	3.02	1.48–6.16	**0.002**	2.49	1.95–3.18	**<0.001**
Round (1/8)	2.83	1.37–5.84	**0.005**	2.90	2.12–3.97	**<0.001**	3.18	2.10–4.83	**<0.001**	3.97	1.96–8.03	**<0.001**	2.88	2.33–3.57	**<0.001**
Round (1/4)	2.24	1.08–4.64	**0.030**	1.81	1.34–2.46	**<0.001**	2.06	1.37–3.11	**0.001**	1.96	0.96–3.99	0.063	2.01	1.63–2.47	**<0.001**
Round (1/2)	1.29	0.60–2.78	0.521	1.38	1.00–1.91	0.052	1.12	0.73–1.71	0.617	1.83	0.85–3.94	0.124	1.37	1.10–1.71	**0.004**
**RANDOM PARTS**															
^τ^00, Judoka	0.679			0.337			0.965			0.947			0.805		
^τ^00, Event	0.000			0.060			0.224			0.035			0.146		
N_Judoka_	244			736			583			544			1,504		
N_Event_	7			36			24			7			88		
ICC_Judoka_	0.171			0.091			0.215			0.222			0.190		
ICC_Event_	0.000			0.016			0.050			0.008			0.034		
Observations	872			3,806			3,165			1,606			9,041		
AIC	1,038.72			4,796.68			3,766.68			2,021.84			11,235.38		
Deviance	873.88			4,371.28			3,146.49			1,613.47			9,678.73		

*For fixed effects, we report the odds ratio [with its 95% confidence interval and significance level (p < 0.05) shown in bold]. Shown are the results for the women's division*.

## Discussion

The goal of our study was to test the hypothesis that blue coloration confers an advantage over white coloration in international judo contests. Many studies on color effects on winning in sports are based on correlational data, which often suffer from several potentially confounding variables explaining the observed color bias in contest success (e.g., seeding system, differences in prior experience). We took advantage of a color assignment change in international judo tournaments creating a unique “experimental” situation. In judo, the first called athlete for the fight used to wear the blue *judogi* and the second called athlete the white *judogi*. However, starting at the World Championships in 2011 (WC 2011), the first called athlete wears the white *judogi* to reflect the importance of the traditional white *judogi*. Since everything else remained the same, we were able to isolate the effect of color of the *judogi* on winning by comparing the win bias before and after this change.

Our analyses support our hypothesis that color does not influence winning in judo. In both the men's and women's division, the winning bias of the first called athlete is strongest in the 1/16 and the 1/8 round and diminishes toward the final, possibly as a result of decreasing asymmetry in athlete ability and hence a diminishing effect of athlete ability—first called athlete association as the tournament progresses. This pattern was observed both before (first athlete blue) and after (first athlete white) the World Championships of 2011 (Figure 2). However, both before and after the switch of the color of the *judogi*, there was still a winning bias for the first called athlete in the final (Table 2). This is in contrast to Dijkstra and Preenen's (2008) study who reported that the effects of the seeding system and asymmetries in recovery times are absent in the final. The explanation for the discrepancy between these studies most likely lies in changes to the seeding system since the semi-finals are still scheduled simultaneously in all tournaments analyzed. The 2008 study was based on 72 tournaments between 1996 and 2005. In this time period, the seeding system often placed seeded athletes randomly across the four pools. By contrast, in more recent tournaments (>year 2007) the highest-ranked athlete was often placed at the top of pool A, causing the association between athlete ability and first called athlete to persist to the final (see Figure 1 in Dijkstra and Preenen, 2008).

**Table 2:** Comparing the number of wins and losses of first called athletes when the color of the first called athlete was blue or white for both the men's and women's division.

**Round**	**Gender**	**First called athlete blue**		**First called athlete white**	
		**χ2**	***P***	**χ2**	***p***
1/32	Men	20.8	**<0.001**	1.05	0.305
1/16		437.38	**<0.001**	219.62	**<0.001**
1/8		440.39	**<0.001**	532.04	**<0.001**
1/4		101.93	**<0.001**	203.63	**<0.001**
1/2		31.19	**<0.001**	36.48	**<0.001**
Final		6.22	**0.013**	3.41	0.0647
1/32	Women	9.59	**0.002**	2.66	0.103
1/16		308.33	**<0.001**	146.14	**<0.001**
1/8		564.55	**<0.001**	532.08	**<0.001**
1/4		214.78	**<0.001**	264.94	**<0.001**
1/2		68.51	**<0.001**	63.71	**<0.001**
Final		24.99	**<0.001**	12.33	**0.005**

*Statistical tests are based on χ^2^ tests. Significant effects are shown in bold. Percentage of wins of first called athlete can be found in Figure 2*.

It has been suggested that uniform color can unconsciously bias the referee's evaluation of identical performances. Evidence for such a psychological effect in referees has been found in team wearing black uniforms (Frank and Gilovich, 1988 but see Tiryaki, 2005) or in red vs. blue/green pairings (Hagemann et al., 2008; Krenn, 2015). For example, using color manipulations of photos showing two athletes competing and a large pool of judges, Krenn (2015) found that in two out of three combat sports, athlete's in red were judged to be more aggressive and more likely to win than athletes wearing blue or green uniforms. Athletes in green were judged fairer in boxing and wrestling than athletes wearing red, but there was no effect of blue in either direction. The lack of an effect of blue *judogi* on winning in the present study also argues against the possibility that referees evaluate athletes dressed in blue more favorably than those dressed in white.

Compared to what we know about the effect of blue and winning, the evidence for an effect of red uniform color on winning is substantial for both combat (Hill and Barton, 2005) and team sports (Attrill et al., 2008; Piatti et al., 2012; but see Allen and Jones, 2014). It is important to note that these studies are retrospective and lack an experimental approach. However, in contrast to the presumed effect of blue, the effects of “red” on winning are theoretically and empirically well-grounded in both the psychological and behavioral ecology literature. There is experimental evidence demonstrating that red coloration itself confers a social dominance advantage in a number of animal species, including human primates (Baube, 1997; Pryke et al., 2001; Dijkstra et al., 2005; Khan et al., 2011). Redness has an inherently intimidating effect on opponents and the behavioral ecology literature suggests that the avoidance of red opponents appears to be innate and not a learned response (Dijkstra et al., 2005; Pryke, 2009). In addition, wearing red is linked to higher heart rate, a higher pre-contest strength and higher testosterone levels in humans (Dreiskaemper et al., 2013; Farrelly et al., 2013). These observations are consistent with the effect of redness on psychological functioning (Ten Velden et al., 2012; Elliot and Maier, 2014) and may explain the effect of red on winning (Wiedemann et al., 2012). There is no evidence that blue coloration impairs psychological functioning or that wearing blue alters physical parameters associated with winning. In the behavioral ecology literature, there are some example of blue phenotypes or morphs winning more territorial disputes but this is the result of blue morphs being inherently more aggressive than their non-blue counterparts, rather than an effect of blue coloration *per se* on the “wearer” or the opponent (Taylor et al., 2016).

In spite of analyzing over 45,000 contests from 162 tournaments, our analysis is still somewhat limited in that it had no purely experimental approach (although one could argue that the color switch can be considered an experimental manipulation, unique in the history of judo). As noted earlier, the switch of the color of the *judogi* of the first called athlete occurred at the WC 2011, and color of the first called athlete was not randomly assigned across years. However, potential order effects are an unlikely explanation for our findings because all contest policies in international judo, including assignment of seeded athletes on the contest sheet, remained the same. This means that biases between first and second called athletes arising from the seeding system, prior experience, and intercontest interval were exactly the same before and after the *judogi* color switch.

Our results suggest, in contrast to previous claims (Rowe et al., 2005; Matsumoto et al., 2007; Sorokowski and Szmajke, 2011; Julio et al., 2015), that no effect of blue judo outfit on winning exists. A win bias for blue was found in the 2004 Athens Olympic Games (Rowe et al., 2005) but their study was confounded by at least four factors arising from the seeding system and asymmetries in prior experience (Dijkstra and Preenen, 2008). A win bias for blue was also observed in randomly selected matches in a judo competition in Brazil with no seeding system (Julio et al., 2015). However, in this study asymmetries in prior experience regarding outcome of previous fights and recovery times remained potential confounding factors as explained in Dijkstra and Preenen (2008). Matsumoto et al. (2007) found a strong blue bias in winning in four major international tournaments (World Championships of 2001, 2003, 2005, and the 2004 Athens Olympic Games). It is surprising that five major tournaments (World Championships of 1999, 2000, 2002, 2004, and the 2000 Sydney Olympic Games) in almost the same time frame with no or the reverse bias were not reported in this study. Future studies should rely on more extensive datasets and researchers should make themselves familiar with the context within which contests take place in international judo tournaments to identify potential confounding factors (Dijkstra and Preenen, 2008).

Overall, our analysis suggests that the blue-white outfit pairings do not affect contest outcomes, consistent with an early study (Dijkstra and Preenen, 2008). The lack of an effect of blue is also consistent with the observation that red but not blue has repeatedly been shown to influence psychological of physiological functioning in both opponents and those wearing red uniforms (Wiedemann et al., 2012, 2015; Dreiskaemper et al., 2013; Krenn, 2015). Research on color biases in winning may contribute to improving fairness in sport and equal opportunities for each athlete, regardless of their uniform color. For example, previous studies have stated that “smart use of color may improve team results” (Olde Rikkert et al., 2015). Our findings may have important implications for (combat) sports policy makers; as our results suggest, a blue–white pairing ensures an equal level of play. Moreover, we urge color psychology and sports researchers to thoroughly investigate alternative explanations and use solid and extensive datasets for examining color effects in sports.

## Author contributions

PD: Analyzed the data and wrote the manuscript; PP: Wrote the manuscript; HvE: Collected the data, entered the data, analyzed the data, and contributed to the writing of the manuscript.

### Conflict of interest statement

Author HvE is employed by Judoinside.com. The other authors declare that the research was conducted in the absence of any commercial or financial relationships that could be construed as a potential conflict of interest.
